# High Genetic Diversity and Structure of *Colletotrichum gloeosporioides* s.l. in the Archipelago of Lesser Antilles

**DOI:** 10.3390/jof9060619

**Published:** 2023-05-27

**Authors:** Pauline Dentika, Jean-Marc Blazy, Angela Alleyne, Dalila Petro, Anderson Eversley, Laurent Penet

**Affiliations:** 1 Institut National de Recherche Pour L’Agriculture, L’Alimentation et L’Environnement (INRAE), Research Unit ASTRO, F-97170 Petit-Bourg, Guadeloupe, France; 2 Department of Biological and Chemical Sciences, Faculty of Science and Technology, Cave Hill Campus, University of the West Indies, Bridgetown BB11000, Barbados; 3 Barbados Agricultural Management Company, Warrens BB23028, Barbados

**Keywords:** *Colletotrichum gloeosporioides* complex, Caribbean, biogeography, yam anthracnose, archipelago, pathogen dispersal

## Abstract

*Colletotrichum gloeosporioides* is a species complex of agricultural importance as it causes anthracnose disease on many crop species worldwide, and strong impact regionally on Water Yam (*Dioscorea alata*) in the Caribbean. In this study, we conducted a genetic analysis of the fungi complex in three islands of the Lesser Antilles—Guadeloupe (Basse Terre, Grande Terre and Marie Galante), Martinique and Barbados. We specifically sampled yam fields and assessed the genetic diversity of strains with four microsatellite markers. We found a very high genetic diversity of all strains on each island, and intermediate to strong levels of genetic structure between islands. Migration rates were quite diverse either within (local dispersal) or between islands (long-distance dispersal), suggesting important roles of vegetation and climate as local barriers, and winds as an important factor in long-distance migration. Three distinct genetic clusters highlighted different species entities, though there was also evidence of frequent intermediates between two clusters, suggesting recurrent recombination between putative species. Together, these results demonstrated asymmetries in gene flow both between islands and clusters, and suggested the need for new approaches to anthracnose disease risk control at a regional level.

## 1. Introduction

*Colletotrichum* is a widespread pathogen of cultivated plants [1], causing anthracnose disease or fruit rot or stem dieback on many crops worldwide [2,3,4]. Its ubiquity in both wild [5] and cultivated environments [6] is probably increased by its relatively complex ecology, with lifestyles ranging from casual commensal endophyte [7] to parasitic pathogen, biotrophic to necrotrophic phases [8] and organizing as multiple species complexes [9] with blurring degrees of gene flow and varying levels of host ranges and agressivity on their incipient hosts [10,11,12]. It has long been regarded through the lens of pathogen–host interaction pairs, with a transient historical redefinition as morpho-species complexes (formally changing recognized species from the thousands down to twelve clearly identified morphs [13]). Current classification trends are building on bar-code-like sequence approaches to systematic [1,14,15], and identified species are reformulated progressively and their total number is increasing back (currently within the hundreds) [16]. Many issues regarding characterizing the species at more ecologically relevant levels remain, and these might at least partially be assessed via regular population genetic analysis and identifying polymorphisms segregation pools both at local and regional scales.

Studies of the genetic structure of populations have shown a fairly high dependency on the kind of marker in use. In this regard, studies of populations of *Colletotrichum gloeosporioides* did not escape this technological pitfall: early studies with isozymes demonstrated a quasi lack of structure within hosts and stronger albeit still low levels between strains sampled on different hosts [17], thus feeding the prevalent view of species as coupled host–pathogen pairs. With markers progressively increasing the fine-scale definition of polymorphisms (e.g., [18,19,20,21]), greater levels of population structure was consequently described, suggesting the prevalence of panmixy and admixing [22,23], sometimes the structure with low migration between populations [24] over large geographical scales [25] before the signature of isolation by distance is met (but see [26]). It is also documented that the local diversity is associated with breeding programs and lacking in wild hosts (e.g., [27]). Overall, populations of the species *C. gloeosporioides* demonstratingly behave as huge panmictic units with few barriers to gene flow, as studies over long-distance dispersal also suggest [26].

In contrast, many biological processes are susceptible to very local influences, especially for generalist fungi with important asexual multiplication, from clonal selection to competition [6] or biases in host response and even vegetation structure [28]. While long-distance dispersal with wind allows for huge genetic admixture [29,30], reliance on rain dispersal at a local scale via conidia in *Colletotrichum* deeply changes the nature of pathogen propagation [31], with most conidia only spreading up to within a meter from the initial necrosis [32]. Vegetation might thus behave as a propagation filter, either as a direct barrier, or as differentially allowing for strain dispersal if asymmetry exists in asexual propagule multiplication based on host strain affinity. This might eventually also affect global disease prevalence, as suggested for example by producers strategical shift from more sensitive species and varieties to less susceptible ones [33] or adoption of varietal admixtures as more resilient [34]. In traditional agricultural landscapes dominated by the mosaic of fields with very different, diverse and patchy feral and neighbouring [6,35] or even wild floras [5], the nature of pathogen intra-species diversity itself is thus expected to be dramatically high for generalists such as *Colletotrichum*.

Our lab recently developed microsatellite marker sets for *C. gloeosporioides*, with local historical strain records demonstrating high levels of polymorphism [36], confirming previous regional analysis [37]. Given the high-resolution ability of these markers, we investigated the existence of a potentially lower-scale structure of polymorphism in the Lesser Antilles, with a focus on the naturally constraining archipelago structure of the environment. We focused on *C. gloeosporioides* complex s.l. sampled from Yam varieties (*Dioscorea alata* L.) without investigating taxonomic identity beyond testing *C. gloeosporioides*/*C. acutatum* boundaries [38]. Our questions were thus: is *C. gloeosporioides* genetically diverse within the Lesser Antilles geographical context and does geography impact it? What is the influence of migration in *C. gloeosporioides* in the region? Does it organize in distinct genetic clusters/putative species?

## 2. Materials and Methods

From November to December 2015, we collected information on farm management practices and varietal diversity from yam producers and sampled their yam fields for necroses on leaves in four islands of the Lesser Antilles: Barbados, Guadeloupe (with both tropical humid Basse Terre and dry Grande Terre areas considered distinct populations due to climate and altitude contrasts, see [39] for geographic details) and its high farming dependency Marie Galante and Martinique. We collected 15 necrotic yam leaves in sample fields (except for in Barbados, where yam plots were bigger and we increased sampling effort to 25 necrotic leaves for the sake of field size representativeness) during the day and placed them in Eppendorfs filled with 2 mL of autoclaved V8 solutions. In the lab, we rinsed each collected necrosis for 1 min in hypochlorite solution, followed by a 1 min bath in alcohol, before two further rinsing steps of 1 min in distilled water [6]. We then placed necroses on Petri dishes with S media to facilitate the growth of *Colletotrichum* strains. After 5 days, we verified whether fungi belonged to the *C. gloeosporioides* complex based on conidia morphology and placed study strains in V8 liquid culture media for three days at room temperature, then kept microtubes refrigerated at 4 °C for a few days before multiplication and DNA extraction. The prevalence of *C. gloeosporioides* was very diverse in the sample fields, and was on average 48.26% (range 7–88%). In 2016, DNA extractions were conducted from the V8 solutions, using a FastDNA kit (MP Biomedicals, Irvine, CA, USA) using Lysing Matrix A for fungal cell lysis. Beforehand, we amplified via PCR the CaInt2, CgInt and ITS4 region to confirm prior visual assessment by microscopy [38]. Every study strain correctly amplified the expected fragment for *C. gloeosporioides*. Nevertheless, 3 strains also amplified fragments diagnosing *C. acutatum*, and were further dismissed from the study sample. We genotyped the strains for 4 microsatellite loci recently developed in the lab (markers Cg150, Cg68, Cg71, Cg92) [36], with the following forward and reverse primers, respectively: TACCAGGGGTGGCAGCTC and GGTCCAGGGACTCAAGCTC for Cg150, TGGTCTGCTTCTCGACACTG and AGCCAAGAGACCAAGCAAGA for Cg68, TGATGGTTGTCATGGGATTC and GATCATGTCTCCATCCGCTC for Cg71 and CATTTTCCACAGCCCACAC and GCAGCAGGTGTGAGAAGAGA for Cg92. Genotypic stability was verified by randomly retesting with secondary independent PCR amplification. The primers had PCR conditions consisting of a denaturation stage at 95 °C for 5 min followed by 40 cycles at 95 °C for 30 s, 59 °C for 30 s, 72 °C for 30 s (more details in [36]).

The sample size was 560 sample strains in total from 58 yam fields, from 16 fields in Guadeloupe (10.38 ± 6.6 strains per field, range 1–22), 18 fields in Martinique (4.70 ± 3.2 strains per field, range 1–12) and 24 fields in Barbados (11.75 ± 5.5 strains per field, range 2–22). Since there was a dramatic variation in strains sampled by fields, the field was not further analyzed as a structuring level for genetic diversity. Genetic analysis was run with hierfstat [40] in R [41]. We report here allelic diversity at each study locus, population structure indices: H_s_, local gene diversity; H_t_, total gene diversity; H_t_′, gene diversity corrected for sampling size; D_st_, genetic distance among populations; D_st_′, genetic distance among populations, corrected for sampling size; F_st_, indice of structuration; F_st_′, indice of structuration, corrected for sampling size; D_est_, shared genetic diversity among population, or Jost indice. We estimated migration rates with the following formula: Nm = [(1/F_st_) − 1]/2, adapted from Wright [42] by correcting for ploidy level (*C. gloeosporioides* species being haploid, we divided by 2 where 4 is used in diploid populations, see [43]). Lastly, we conducted a principal component analysis on individual genotypes frequencies (centered, unscaled matrix) with hierfstat [40], to explore potential clustering occurring in our dataset.

## 3. Results

### 3.1. Genetic Diversity

**Figure 1:**
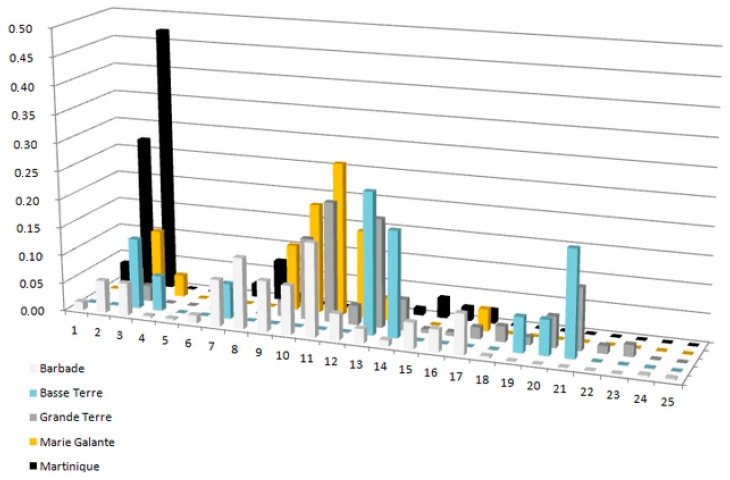
Variation in allelic diversity among study islands. Example from locus Cg150, truncated for alleles over #25 (all are rare alleles mostly from Barbados).

The extant of genetic diversity was very high for all populations, with the four loci demonstrating high allelic richness (42 alleles for Cg150, 50 alleles for Cg68, 54 alleles for Cg71 and 76 alleles for Cg92), a high share of these alleles among islands (see Figure 1, though islands with lower prevalence such as Basse Terre and Marie Galante have lower allelic diversity levels overall) and greater diversity in Barbados in general (Table 1). Most alleles were nevertheless rare (low frequency), resulting in an important number of diagnostic alleles for islands, and rarefied allele estimates averaged between 2 and 3 (Table 1).

As a consequence of this dramatic diversity level, most strains were characterized by a unique genotype, and we counted only 20 multilocus genotypes shared among *Colletotrichum* samples, up to 61 strains in total. Most identical multilocus genotypes occurred in pairs or triplets (mean clonality level = 3.05 ± 1.80, range 2–8). Clonality was thus representing about 10.89% of total samples, spread similarly among islands. Interestingly, few clones were actually sampled within fields (two clones in Barbados, one clone in Basse Terre, two clones in Grande Terre and two clones in Martinique), while many clones were distributed in different fields within populations (a situation found nine times in Barbados, three times in Basse Terre, three times in Grande Terre and three times in Martinique). In a few cases, clones we sampled between different populations: three times between fields in Basse Terre and Grande Terre, and once between Grande Terre and Barbados. The latter situations probably represented recent migration events.

Since allelic diversity was shared among populations, with the exception of diagnostic alleles, and actually reached reasonably high levels everywhere, all loci had important impacts on the structuration of genetic diversity in the Archipelago (local allelic richness was important, but always lower than expected as a single theoretical panmictic population: H_s_ was lower than H_t_ or H_t_′, and both D_st_ and D_st_′ show an important share variation between populations for all loci, Table 2). As a result, both F_st_, F_st_′ and Jost D_est_ estimates give evidence of a geographical effect of Archipelago condition, with signs of moderate to strong genetic structuring of *C. gloeosporioides* (Table 2).

### 3.2. Estimates of Migration and Gene Flow

Since there was overall evidence of genetic structure in the islands (Table 2), we calculated pairwise F_st_ values between study populations. There was indeed variation in the extant of genetic structuration between islands (Table 3), and interestingly, the differences were not consistent with geographic distance: for example, Barbados demonstrated smaller values with Grande Terre and Marie Galante than with closer Martinique. Alternately, geographically close populations had greater values (example: Basse Terre and Basse Terre, Table 3). Lastly, both Grande Terre and Marie Galante populations hinted to behaving as a single panmictic population with recurrent propagule exchange.

**Figure 2:**
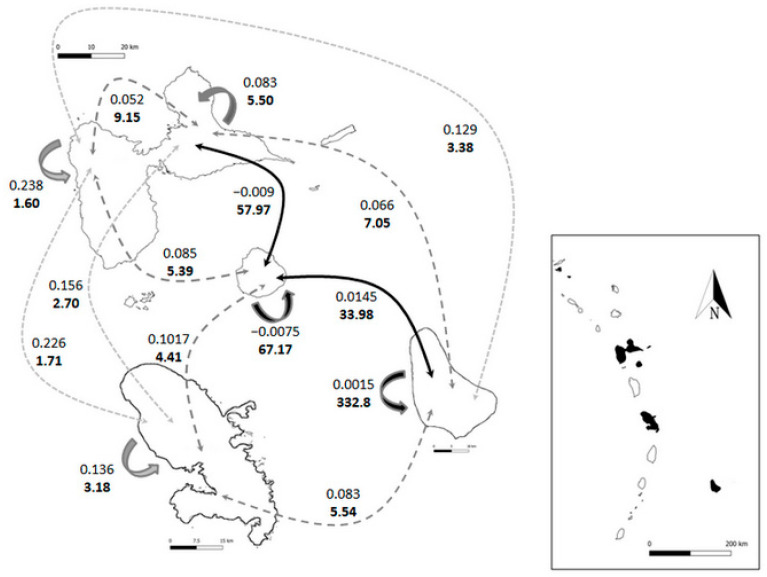
Gene flow for *Colletotrichum gloeosporioides* among study islands. Upper values represent pairwise F_st_ estimates between populations. Lower values are estimated annual number of migrant spores among islands. Flows are encoded both via colour (light grey = low migration rate, dark grey = intermediate migration rate, black = high migration rate) and dash lines (small dash line = low migration rate, medium dash line = intermediate migration rate, plain line = high migration rate). Significant gene flows are thus Barbados to Marie-Galante and Marie Galante to Grande Terre. Auto-arrows represent flow within populations (yam fields within island) and follow the colour code described above. Islands are not following geographic arrangement for the sake of clarity (actual geographic arrangement on the right map). Scale for Guadeloupe (Upper Island) is 20 km, scale for Martinique (lower left) is 15 km, and scale for Barbados (lower right) is 10 km. Islands are grossly at scale comparatively to each other. Scale for the Lesser Antilles is 200 km.

We estimated migration rates based on pairwise F_st_ and values indicated a broad variation in the number of migrating spores both within and between islands (Table 3). These estimates suggest different processes, as migration between islands reflects the long-distance dispersal and relative contribution of other genetic pools to local gene admixtures, while migration estimates within islands reflect the ease with which spores can establish via local dispersal, operating through altitude, climatic and vegetation constraints. Estimating the average number of spores contributing to genetics and mating pools allowed us to envision how gene flows link the different islands (Figure 2). Some flows are indeed much lower than others, and there were strong asymmetries in the contribution of migration between islands.

Overall, the dispersal within islands followed two contrasting trends: situations where local dispersal was lower on average than long-distance migration (Basse Terre especially, but also Grande Terre, and the similar but less marked Martinique), and situations where local dispersal was greater than long-distance migration (Barbados, Marie Galante) (Figure 2). We can safely assume that genetic dynamics for C. gloeosporioides complex in the Lesser Antilles follow a metapopulation pattern with both source and sinks of strains. Since the pattern does not reflect the physical distance between islands (and does not hint at isolation by distance), alternative hypotheses need to be developed, among which climate and vegetation act as a local dispersal barrier, and winds as a major driver of gene flow for long-distance dispersal (see discussion).

### 3.3. Genetic Clusters

**Figure 3:**
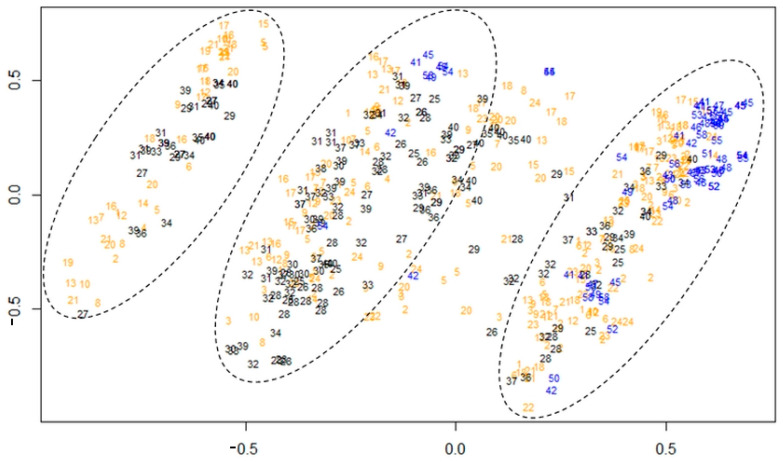
Genetic clusters based on genotypic composition of study strains. Black colour illustrates Guadeloupean strains, blue colour strains from Martinique and orange strains from Barbados. Labels represent sample field, so that different strains may share the same label if they were sampled together in the same field.

Congruently with high levels of dispersal, clustering was not really altered by geography, yet three independent genetic clusters emerged from our data, reflecting three sampled *Colletotrichum* species from *C. gloeosporioides* complex in yam fields in the Caribbean (Figure 3). Preliminary sequence analysis indicates that one of them is *C. siamense*, and a second one is a currently undefined species (S. Guyader, personal communication) (work in progress). All islands presented their share from two clusters (origins are interspersed in both), in approximately similar proportions save for Martinique which demonstrated no samples from the leftward cluster (Figure 3). Interestingly, one cluster (on the left) is separated and stands alone, possibly as a true species and genetically isolated from the other clusters (though this might otherwise be due to lack of sampling), while two clusters seemed interconnected by numerous intermediate strains, strongly suggesting that recombination between strains from both clusters is occurring at high enough frequency.

## 4. Discussion

Our results showed astonishingly high levels of genetic diversity of *C. gloeosporioides* complex sampled on Yam in fields of three Caribbean islands from the Lesser Antilles (Guadeloupe—Basse Terre, Grande Terre, Marie Galante, Martinique and Barbados). Allelic diversity was rich enough to demonstrate both diagnostic alleles, sometimes to field level, and importantly shared genetic components between islands. Clonality was nevertheless relatively low, suggesting asexual multiplication is not contributing strongly to the local structure at the field level, but that contamination occurs via many sources, most probably from local vegetation. Genetic structure was strong, indicating that study populations indeed function as distinct entities at least partially, yet also highlighted the importance of long-distance migration (wind dispersal between distant islands), often with rates greater than local dispersal (suggesting factors such as vegetation and local climate are impeding propagation locally). Lastly, PCA highlighted three distinct genetic clusters, indicative of the sampling of three putative species within the complex, with one cluster fully differentiated while two clusters exhibited numerous intermediate genotypes thus hinting to casual recombination between strains. Clusters were sampled in all the study islands. We will discuss these results in the light of anthracnose disease management on yams.

Genetic diversity levels were high, as expected given the propensity of microsatellite markers to mutate. Furthermore, at field and population levels, allelic diversity was more important than clonality, and most sampled strains had distinct genotypes. This study is confirming earlier results based on RAPD markers in the same patho-system (Yam/*Colletotrichum*) [37] or in other crops [26]. This stands in sharp contrast to most crop diseases, where strains are fairly homogenous, genetically speaking, when epidemics declare regionally (e.g., [44,45,46]). Here, clonality accounted only for approximately 10% of strains, and clones were often sampled as few units (multilocus genotype shared between a few strains only, three on average). Moreover, clones were more often sampled between than within fields (thus confirming the importance of dispersal as a structuring factor for genetics in the species complex, see below). Most importantly, a low level of clonality between strains is indicative of a high prevalence of sexuality and recombination compared to asexual multiplication, despite a high capacity for multiplication via conidia from necroses. The observation would occur if broad strain reservoirs accumulating fungi diversity and local contamination dynamics co-occur, which seems to be the case with *Colletotrichum* as prevalence in natural flora was shown to be particularly high [5]. This pattern of diversity is at odds with most fungal diseases, where pathogenic strains are often genetically homogenous and spread regionally on susceptible cultivars. In our case, the genetic pool of strains is highly diverse, and as a result, putative aggressive strains can declare new epidemics at any time. We should expect direct consequences for agriculture, since this means the pool of potentially pathogenic strains is dramatic, and efforts toward pyramiding resistance genes in varietal breeding may be circumvented faster [47], thus reducing the durability of disease management via increased disease resistance. A possible solution to this issue would be carefully planned varietal turnover at a regional level, to reduce local pathogenic load impact and decrease anthracnose risk.

Migration rates were reasonably high, yet varied considerably between constitutive populations, segregating situations where the intra-deme dispersal was lower than long-distance migration, and conversely, situations where local dispersal was greater than migration. Overall, these results suggest strong metapopulation dynamics [48], with some key populations contributing heavily in genetic composition at broader scales (such is the case of Barbados in our study). Monitoring these source populations, especially for strain aggressiveness, may be an important strategy in disease control and management [49]. Long-distance dispersal was shown to occur in the region (Mexico to Trinidad, see [30]), and dispersal may not. Our results suggest intra-population dispersal may be fairly low: the population of Basse Terre has the lowest local dispersal, for example. This population is geographically characterized by denser tropical humid and altitude vegetation, possibly implying that forested vegetation increase the viscosity of the landscape in terms of spore dispersal (trees as spore traps hypothesis [50]), or increased local adaptation requirements compared to drier areas, or both. If this hypothesis holds scrutiny, then a simple disease control strategy might be to increase recourse to trees in agriculture, for example planting more hedges, and even the field if vegetation margins can become inoculum sources following fungi establishment [35]. Lastly, long-distance dispersal is an important driver of the system. The Caribbean region is subjected to hurricane seasonality (during the rainy season), so that *Colletotrichum* species may be seen as “storm riders” and following dominant winds (northwards) as migration roads. This reinforces the importance of monitoring source populations for disease risk estimation. A further hypothesis regarding wind-based long-distance dispersal, not accounted for in the case of anthracnose to the best of our knowledge, is that the Caribbean region is also casually and seasonally subjected to sand mists originating from Sahelian West Africa (during the dry season, or Lent) [51]. Since sand mists are known to help fungal spores travel in addition to sand [52], West Africa could be another region contributing to the genetics of *Colletotrichum* species in the Caribbean, and this phenomenon should be the focus of further research, especially focusing on Ivory Coast where *D. alata* is also the dominant yam cultivated as in the Caribbean islands [53]. In summary, long-distance dispersal is a very important component of anthracnose dynamics [30], and can possibly jeopardize management and control practices. Possible solutions may involve creating agriculture environments with decreased dispersal, such as greater recourse to hedges and forested areas.

Principal component analysis yielded three genetic clusters representing putative species on yams, all grossly distributed in sampled islands, and broadly coexisting locally at field level, though one species seemed not sampled in Martinique. Interestingly, one of these clusters is standing apart, while the two others show signs of genetic admixing and recombination for a significant number of sample strains. It is worth noticing that *Colletotrichum* spp. are known to casually recombine [54,55] and that species delineation, as in other fungi, is sometimes a blurry concept. In our initial dataset, three strains amplified both fragments allegedly delineating two species complexes (*C. gloeosporioides* and *C. acutatum*) [55], though both are known to be closely related and are sometimes a source of taxonomic confusion if the shape of conidia is the only criterion. Here, our results show that recombination might be more frequent between putative species within complexes (as an approximation, 40/560~7.14%, nearly the same level as clonality in the study sample) *Colletotrichum* species are indeed notoriously hard to define, and while the approach of morpho-species developed by von Arx [13] allows gross delineation of complexes, current standing involves sequencing to reach ‘adequate’ taxonomic evaluation. Our results nevertheless suggest that none of morpho-species and sequencing approaches [56] would be fair enough to delimit real species entities, and will be either too liberal (morpho-species line) or possibly too conservative (sequencing/barcoding) in assessing the real diversity of *Colletotrichum* species (and therefore, overestimate diversity in the Genus). Our team usually favours a morpho-species approach to understand the ecology of *C. gloeosporioides* species complex (e.g., [5,6,36]), and we thus call for more flexibility and inclusion of a diversity of stances and viewpoints regarding the complex issue of *Colletotrichum* genus worldwide. Evaluating the frequency of recombination events both within species complexes and between species complexes is a promising avenue of research in our quest to understand the biology of these important crop pests.

## 5. Conclusions

Strains from the *C. gloeosporioides* complex sampled in Water Yam fields in the Lesser Antilles were genetically highly diverse and demonstrated a dominance of sexual reproduction over clonality and asexual multiplication. Lesser Antilles populations are structured, with important long-distance migration, viscosity in local dispersal probably due to vegetation acting as natural barriers. Some populations (Barbados) are propagule sources at a regional scale. Three species coexist on Yams, but there is strong evidence of recombination between some of them, furthering the importance of sex events in the dynamics of recombination in the Genus and increasing diversity in rich reservoir pools, thus raising anthracnose disease risk. Potential metapopulation functioning in the Caribbean suggests that anthracnose control will be difficult to sustain only by increasing genetic resistance in varieties, though potential solutions exist to manage risk include: i/careful monitoring of strain skill in inoculating yams aggressively, especially in source populations; ii/increasing viscosity of dispersal in the landscape by increasing vegetation/tree cover; and iii/a regional varietal scheme allowing rotation of cultivars with different resistance levels to avoid local matching of *Colletotrichum* strains and yams.

## Figures and Tables

**Table 1:** Summary statistics for allelic diversity and genetic structure among study islands. Number of alleles (A), number of diagnostic alleles (not shared in other islands) (D) and rarefied allele numbers (R) are indicated for each study locus. F_st_ (*p*-values of testing difference from 0 as superscripts) and confidence intervals (95% CI) are produced. Populations behaving as panmictic locally (95% CI includes 0) are indicated in bold. We give statistics for both Guadeloupe globally, or each Guadeloupean area individually (Basse-Terre, Grande-Terre and Marie Galante). Global F_st_ value is 0.095 with 95% CI [0.0285–0.168]. ^NS^ indicate non-significant departure from 0.

	Cg150 (42 Alleles Total)			Cg68 (50 Alleles Total)			Cg71 (54 Alleles Total)			Cg92 (76 Alleles Total)			F_st_	95% CI
	A	D	R	A	D	R	A	D	R	A	D	R		
**Barbados**	33	17	2.76	41	26	2.77	35	24	2.80	61	37	2.79	**0.0016** ^NS^	**−0.0425–0.0232**
Guadeloupe	22	8	2.66	20	8	2.61	12	4	2.20	37	13	2.81	0.1606 ^<0.001^	0.1010–0.2424
Basse Terre	8	0	2.69	6	0	2.54	2	0	1.25	10	0	2.78	0.2381 ^<0.001^	0.0385–0.5275
Grande Terre	20	5	2.66	18	7	2.48	11	2	2.35	35	9	2.86	0.0832 ^NS^	0.0136–0.1387
**Marie Galante**	9	0	2.62	5	0	2.8	3	1	3.00	10	1	2.79	**−0.0745** ^NS^	**−0.1022–0.0610**
Martinique	12	1	2.23	6	0	2.54	22	13	2.84	11	1	2.25	0.1357 ^<0.001^	0.0174–0.2492

**Table 2:** Population structure statistics by locus. H_o_ and F_is_ cannot be documented for haploid species.

	H_s_	H_t_	H_t_′	D_st_	D_st_′	F_st_	F_st_′	D_est_
cg150	0.852	0.922	0.940	0.070	0.088	0.076	0.093	0.593
cg68	0.866	0.925	0.940	0.059	0.074	0.064	0.079	0.550
cg71	0.752	0.850	0.874	0.098	0.122	0.115	0.140	0.493
cg92	0.889	0.937	0.950	0.048	0.060	0.052	0.064	0.545
Overall	0.840	0.909	0.926	0.069	0.086	0.076	0.093	0.537

**Table 3:** Pairwise F_st_ values between study populations, and corresponding migration estimates. F_st_ values below diagonal, and migration estimates above diagonal and in bold. The diagonal indicates intra-population values for both F_st_ and migration between fields within islands. Overall population structure was significantly different from 0 (*p* > 0.001).

	Barbados	Guadeloupe			Martinique
		Basse Terre	Grande Terre	Marie Galante	
Barbados	**332.8** 0.0015	**3.38**	**7.05**	**33.98**	**5.54**
Basse Terre	0.129	**1.60** 0.238	**9.15**	**5.39**	**1.71**
Grande Terre	0.066	0.052	**5.50** 0.083	**57.97**	**2.70**
Marie Galante	0.0145	0.085	−0.0087	**67.17** −0.0075	**4.41**
Martinique	0.083	0.226	0.156	0.102	**3.18** 0.136

## Data Availability

Data are available on request from the corresponding author.
